# Sperm protein 17 is highly expressed in endometrial and cervical cancers

**DOI:** 10.1186/1471-2407-10-429

**Published:** 2010-08-16

**Authors:** Fang-qiu Li, Qun Liu, Yan-ling Han, Bo Wu, Hong-lin Yin

**Affiliations:** 1 Laboratory of Molecular Biology, Institute of Medical Laboratory Sciences, Jinling Hospital, School of Medicine, Nanjing University, Nanjing 210002, China; 2 Department of Pathology, Jinling Hospital, School of Medicine, Nanjing University, Nanjing 210002, China

## Abstract

**Background:**

Sperm protein 17 (Sp17) is a highly conserved mammalian protein in the testis and spermatozoa and has been characterized as a tumor-associated antigen in a variety of human malignancies. Many studies have examined the role of Sp17 in tumorigenesis and the migration of malignant cells. It has been proposed as a useful target for tumor-vaccine strategies and a novel marker to define tumor subsets and predict drug response. This study aimed to investigate the expression of Sp17 in endometrial and cervical cancer specimens, its possible correlation with the pathological characteristics, and its value in the diagnosis and immunotherapy of the related cancers.

**Methods:**

The monoclonal antibodies against human Sp17 were produced as reagents for the analysis and immunohistochemistry was used to study two major kinds of paraffin-embedded gynecological cancer specimens, including 50 cases of endometrial cancer (44 adenous and 6 adenosquamous) and 31 cases of cervical cancer (15 adenous and 16 squamous). Normal peripheral endometrial and cervical tissues were used as controls.

**Results:**

Sp17 was found in 66% (33/50) of the patients with endometrial cancer and 61% (19/31) of those with cervical cancer. Its expression was found in a heterogeneous pattern in the cancer tissues. The expression was not correlated with the histological subtype and grade of malignancy, but the staining patterns were different in endometrial and cervical cancers. The hyperplastic glands were positive for Sp17 in the normal peripheral endometrial and cervical tissues in 10% (8/81) of the patients.

**Conclusions:**

Sp17 is highly expressed in human endometrial and cervical cancers in a heterogeneous pattern. Although the expression frequency of Sp17 is not correlated with the histological subtype, the staining pattern may help to define endometrial and cervical cancers. Sp17 targeted immunotherapy of tumors needs more accurate validation.

## Background

Endometrial cancer and cervical cancer are two of the most common malignancies among females globally [1]. Some of them have a poor prognosis due to their chemoresistance and early metastasis. No specific molecular markers are currently available for the early diagnosis and immunotherapy of these aggressive malignancies [2]. Therefore, there is an urgent need to identify tumor antigens associated with chemoresistance and early metastasis which can then be used as suitable targets for immunotherapy.

The immunogenic protein, Sperm protein 17 (Sp17), is a member of the cancer testis antigen (CTA) family and has been extensively characterized [3-12]. Sp17 is a highly conserved mammalian protein in the testis and spermatozoa of humans and animals including rabbits, mice, baboons, and macaques [13-15]. Human Sp17 has 151 amino acids and an apparent molecular mass of 24.5 kDa [3]. It is comprised of 3 different domains: an N-terminal domain that has 45% homology to a type II regulatory subunit of protein kinase A-anchoring protein, a central domain that contains a sulfated- carbohydrate-binding domain, and a C-terminal domain that has 43% homology to a Ca^2+^-calmodulin-binding domain [4,16]. The function of Sp17 is not yet completely understood; it is thought to potentially play a role in regulating sperm maturation, capacitation, acrosomal reaction, and interactions with the oocyte zona pellucida during the fertilization process [17,18].

The expression of Sp17 in malignant cells was first discovered by Dong *et al *[19] who found the mouse homologue of Sp17 to be highly expressed in metastatic cell lines derived from a murine model of squamous cell carcinoma but not in the nonmetastatic parental line. Various works have demonstrated the aberrant expression of Sp17 in cancers of unrelated histological origin, including multiple myeloma, ovarian cancer, nervous system tumors and esophageal squamous cell cancer [[6,7], and [10]]. A possible role for Sp17 in cancer was demonstrated in transformed lymphoid and hematopoietic cells. As Sp17 mediates cell adhesion and interaction, it was thought to be involved in the migration of malignant cells [18,20]. Other authors and our results demonstrated that its overexpression decreased the chemosensitivity of ovarian cancer cells *in vitro *[20,21]. Moreover, Bumm *et al *showed that Sp17 could be used as a means of discriminating between 2 subsets of primary esthesioneuroblastomas [22].

Human Sp17 was thought to be expressed at low levels in normal tissues other than the testis. Zhang *et al *[9] used a combination of real time PCR and immunohistochemistry to investigate the distribution of Sp17 on a large panel of normal tissues and demonstrated the restricted normal tissue expression of Sp17. Their results showed that although Sp17 transcripts could be detected in some normal tissues by PCR, the levels of expression were <2% of those in normal testis. Sp17 protein was detected only in the testis but not in any other normal tissues by immunohistochemistry with two Sp17 murine monoclonal antibodies, each directed at a non-overlapping B-cell epitope. Thus, the immunogenicity and restricted expression in normal tissues made Sp17 an attractive molecule for the immunotherapeutic procedure of associated cancers [23-26].

In a phase I study, Sp17-pulsed dendritic cells in Sp17+ cancer patients were shown to kill HLA-matched tumor cell lines and fresh tumor cells presenting Sp17 epitopes. In addition, treatment with cytotoxic T lymphocytes (CTL) did not show any apparent side effects in Sp17+ tumor animal models [27]. These findings have implications for the translational development of safe and effective T-cell-based therapies for human ovarian cancer. Gynecological cancers would be the best candidates for Sp17 targeted immunotherapy, since Sp17 expression may be lowest in women.

Other investigators, however, propose that the tissue distribution of Sp17 in humans is more complex than originally thought. Sp17 has been found in human somatic cells, such as the ciliated epithelia of the respiratory airways and both male and female reproductive systems [28], synoviocytes of patients with rheumatoid arthritis [29], and in the melanophages of cutaneous melanocytic lesions [30]. These findings have raised a question as to whether Sp17 may be a useful target for tumor immunotherapy.

We found in our previous work that Sp17 was aberrantly expressed in 43% of human epithelial ovarian cancer tissues [20], but its presence in two other gynecological cancers, endometrial and cervical has not been examined. The aim of this study was to investigate the frequency of the Sp17 expression at the protein level and its cell distribution and localization in various histological subtypes of endometrial and cervical cancers and to analyze its utility as a target for tumor diagnosis and antigen-specific immunotherapy in these patients.

## Methods

### Patient Specimens

A panel of formalin-fixed paraffin-embedded gynecological tumor specimens was obtained from the archival resource of the Department of Pathology of Jinling Hospital, which included 50 cases of endometrial cancer (EMC) and 31 cases of cervical cancer (Table 1). The positive control samples were collected from the testis tissue of an elderly prostatic cancer patient and from the ejaculated spermatozoa of patients at semen examination. All tumors were classified according to the WHO criteria. All data, including the age of the patient and histological type and grade of the tumor, were obtained from pathological records. To determine whether Sp17 was expressed at the protein level in non-pathological tissues another 81 specimens were taken from the periphery of the lesions. The absence of pathological cells or tissues was subsequently confirmed by microscopy. The study protocol was approved by the Ethics Committee of the Hospital and informed consent was obtained from all patients included in the study.

**Table 1:** Characteristics of the patients

Tumor histological subtype	Patients, *n*	Mean age (range), yr	Positive, *n *(%)
Endometrial cancer	50	54 (34-71)	33 (66.0)
Adenocancer	44	54 (34-71)	29 (66.0)
Adenosquamous cancer	6	49 (34-57)	4 (66.7)
Cervical cancer	31	46 (17-72)	19 (61.3)
Adenocancer	15	40 (26-56)	7 (46.7)
Squamous cancer	16	50 (17-72)	12 (75.0)

### Generation of Sp17 Monoclonal Antibodies

Recombinant Sp17 and its monoclonal antibodies were produced as previously described [31]. Briefly, the nucleotide sequence encoding Sp17 was amplified from human testicular RNA and cloned in pET-28a(+) (Novagen) containing an N-terminal 6-histidine fusion tag. Proteins were expressed in *Escherichia coli *BL21 (DE3). After lysis of the bacteria Sp17 was purified via nickel affinity chromatography. The recombinant Sp17 protein was used to immunize BALB/c mice for generating monoclonal antibody-producing hybridomas. The hybridoma supernatants were screened by ELISA for the presence of antibodies and the hybridoma cell lines producing antibodies against the recombinant Sp17 protein were cloned. The specificity of these antibodies was then confirmed by immunohistochemistry of natural Sp17 from the human testis and ejaculated spermatozoa. Monoclonal antibodies were purified from hybridoma ascites using a HiTrap Protein G HP affinity column (Amersham Biosciences).

### Immunohistochemistry

Tissue sections (3 μm) were placed on glass slides, heated at 60°C for 20 min, and then deparaffinized with xylene and ethanol. For antigen retrieval, tumor specimens mounted on glass slides were immersed in preheated antigen retrieval solution (DAKO high pH solution; DAKO) for 20 min and cooled for another 20 min at room temperature. After the inactivation of endogenous peroxidase, 2 μg/ml 3C12 antibody was added and incubated overnight at 4°C. The primary antibody was detected with HRP-anti-mouse IgG (DAKO). Diaminobenzidine (DAB) substrate was added for 7 min followed by washing with deionized water, and hematoxylin was applied for 1 min to counterstain the tissue sections. The tissue sections were dehydrated with graded ethanol followed by xylene and the coverslips were attached with permount. The immunohistochemical reaction was evaluated in 5 areas of the slide sections under the light microscope at 40× and 100× objective magnifications and semi-quantitatively graded by two expert pathologists. The extent of immunohistochemical reactivity was graded as follows: negative; +, 5-25% of the cells stained; + +, >25-50% of the cells stained; + + +, >50-75% of the cells stained; and + + + +, >75% of the cells stained. The negative control slides omitting the primary antibody were included in all assays. The cancer specificity of the binding of the Sp17 antibody was confirmed in competitive binding assays using soluble recombinant Sp17 protein.

### Statistical Analysis

All statistical analyses were performed with the SPSS software. Differences in proportions were evaluated by Fisher's exact test as appropriate. Statistical correlations between Sp17 expression and pathological parameters (such as the histological subtype and grade of tumor) were calculated using the Kruskal-Wallis test. Results were considered statistically significant at the *P *< 0.05 level.

## Results

### Production of Recombinant Sp17 Protein and Its Monoclonal Antibodies

**Figure 1:**
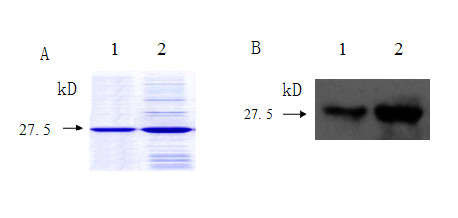
**Production of recombinant human Sp17 and specific identification of hSp17 mAbs by Western blot**. *A*. SDS-PAGE analysis of recombinant human Sp17; *B*. Specific identification of hSp17 mAb 3C12 by Western blot. Protein from human testis (*B1*) and ejaculated spermatozoa (*B2*).

**Figure 2:**
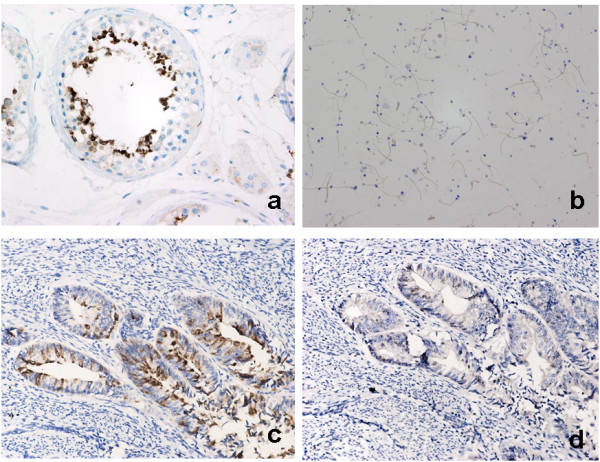
**Immunohistochemical characteristics of the Sp17 monoclonal antibody**. *a*. testis from an old patient with prostatic cancer; *b*. ejaculated spermatozoa; *c*. EMC staining before blocking; *d*. EMC staining after blocking.

Successful production of recombinant Sp17 was demonstrated by SDS-PAGE, which showed a protein of the expected size of around 30 kDa. Following passage through the Ni-NTA affinity column and numerous rounds of washing the recombinant protein was purified (Fig 1A) and used for generating the monoclonal antibody and testing the specificity of the immunohistochemistry assay. Two hybridoma cell lines (3C12 and 3D6) secreting mAb against Sp17 were developed (Fig 1B). The 3C12 monoclonal antibody exhibited intense immunostaining in the human testis (Fig 2a) and ejaculated spermatozoa (Fig 2b). The specific binding of the Sp17 antibody to EMC cells was further confirmed in a competitive binding (blocking) assay, which showed the abrogation of staining when the Sp17 antibodies were pre-incubated with 100 μg/ml soluble recombinant Sp17 proteins (Fig 2c, 2d).

### Immunohistostaining of Sp17

A total of 81 archival gynecological malignancy specimens were investigated by immunohistochemistry. Positive staining was observed in 52 (64%) of the 81 cancer samples. 66% (33/50) of all the samples in EMC were Sp17 positive; 61% (19/31) in cervical cancer, 47% (7/15) in adenocancer and 75% (12/16) in squamous cancer. Tabs 2 and 3 show the frequencies of Sp17 expression at the protein level in EMC and cervical cancer with different histological types and differentiated degrees. The statistics show no significant differences among all groups. Sp17 expression was also observed in 10% (8/81) of hyperplastic gland cells in peripheral endometrial and cervical tissues.

**Figure 3:**
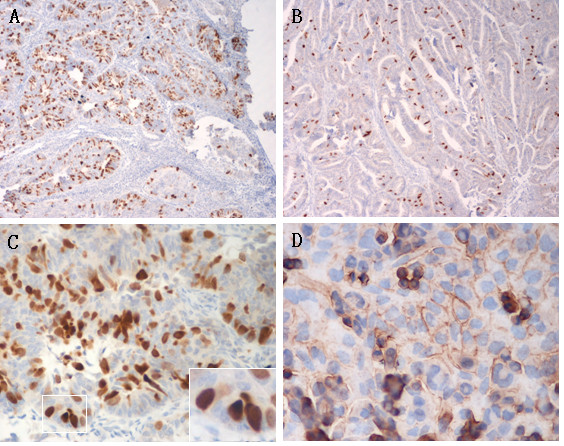
**Immunohistochemistry of Sp17 in the EMC tissues (*A-C*) and cultured cell line (*D*)**. The expression of Sp17 was heterogeneous: *A*. confertim; *B*. scattered. *C*. Sp17 was localized in the cytoplasm and nucleus, with enplaque; *D*. Sp17 was localized in the cytoplasm, nucleus and membrane. (*A, B *10×; *C, D *40× original magnification, 100× inset).

**Figure 4:**
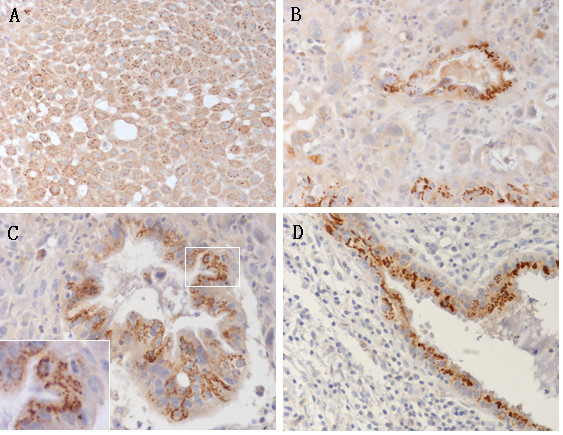
**Immunohistochemistry of Sp17 in cervical cancer tissues (*A-C*) and hyperplastic gland (*D*)**. The staining pattern of Sp17 was granulo in all the original cervical tissues and located in the cavosurface of adenocancer and the hyperplastic gland. *A*. squamous cancer; *B, C*, adenocancer; *D*. hyperplastic glands in the periphery of the lesions. Sp17 was localized in the cytoplasm but had different intensity. (*A, B*, *D*, 20×; *C*, 40× original magnification, 100× inset).

The Sp17 protein was strongly expressed in the adenous, adenosquamous and squamous cancer cells of the epithelial malignancies (Figs 3 and 4). The expression patterns of Sp17 in gynecological tumors were always heterogeneous. The density of Sp17 expression was different from one patient to another. Staining revealed mainly enplaque in endometrial cancers (Fig 3) and granulo in cervical cancers (Fig 4). In keeping with previously reported results [10,11], we found Sp17 mainly localized in the cytoplasm of a variable number of tumor cells with positive staining of the cell nucleus and membrane. To corroborate the immunohistochemical findings the localization of Sp17 was also determined in the cultured ovarian cell line HO8910 which overexpressed Sp17 by DNA transfection in our laboratory [20]. The photomicrograph in Fig 3D shows Sp17 immunoreactivity in the cytoplasm, nucleus, and membrane of HO8910 cells.

## Discussion and Conclusions

To assess the diagnostic and immunotherapeutic utility of Sp17 for gynecological cancers the present comprehensive analysis of Sp17 was undertaken on two kinds of gynecological malignancies. We examined Sp17 expression in tumors by IHC. The results showed the presence of Sp17 in various gynecological epithelia cancers, including 66% (33/50) of EMC and 61% (19/31) of cervical cancer in Chinese patients. The present study is the first to show the expression of Sp17 at the protein level in a high proportion of endometrial cancer and cervical cancers. The results of our study, together with previous findings, show a high positive rate of Sp17 expression in ovarian cancer, in 68% of cells at the mRNA level [11] and 43% of cells at the protein level [20]. These data suggest that Sp17 was highly expressed in gynecological epithelial cancers.

We investigated the distribution of Sp17 in tissues and its immunolocalization in cells and found that it was positive but with different intensity and density in all kinds of gynecological epithelial cancers, including adenous, adenosquamous, and squamous cancers. It was even in a few hyperplastic glands in normal peripheral tissues. Although it had a high total positive rate, Sp17 was expressed in a low density in half of the positive tissues (Tables 2 and 3). In adenocancer and hyperplastic glands Sp17 was located in the cavosurface (Figs 3 and 4). We also found different staining patterns of Sp17, with enplaque in endometrial cancer but granulo in cervical cancer and the cervical hyperplastic gland. This may help to define tumors of the two different origins. Sp17 expression showed no correlation with the histological subtypes and grades of tumors of the same origin.

**Table 2:** Sp17 expression and its correlation with the clinicopathological characteristics of 50 endometrial cancers

Clinicopathological characteristics	Negative	Positive	+	++	+++	++++
All tumors, *n *(%)	17 (34.0)	33 (66.0)	9 (18.0)	7 (14.0)	13 (26.0)	4 (8.0)
Tumor grade, *n *(%)						
G1	4 (8.0)	13 (26.0)	4 (8.0)	1 (2.0)	6 (12.0)	2 (4.0)
G2	11(22.0)	16 (32.0)	2 (4.0)	5 (10.0)	7 (14.0)	2 (4.0)
G3	2 (4.0)	4 (8.0)	3 (6.0)	1 (2.0)		
Histology, *n *(%)						
Adenocancer	15 (30.0)	29 (58.0)	9 (18)	4 (8.0)	12 (24.0)	4 (8.0)
Adenosquamous cancer	2 (4.0)	4 (8.0)		3 (6.0)	1 (2.0)	

-, 5%; +, <25%; ++, 25-50%; +++, 50-75%; ++++ > 75%.

**Table 3:** Sp17 expression and its correlation with the clinicopathological characteristics of 31 cervical cancers

Clinicopathological characteristics	Negative	Positive	+	++	+++	++++
All tumors, *n *(%)	12 (38.7)	19 (61.3)	8 (25.8)	3 (9.7)	7 (22.6)	1 (3.2)
Tumor grade, *n *(%)						
G1	4 (12.9)	5 (16.1)	1 (3.2)	2 (6.5)	2 (6.5)	
G2	4 (12.9)	9 (29.0)	4 (19.4)		4 (12.9)	1 (3.2)
G3	4 (12.9)	5 (16.1)	3 (9.7)	1 (3.2)	1 (3.2)	
Histology, *n *(%)						
Adenocancer	8 (25.8)	7 (22.6)	4 (12.9)		2 (6.5)	1 (3.2)
Squamous cancer	4 (12.9)	12 (38.7)	4 (12.9)	3 (9.7)	5 (16.1)	

-, 5%; +, < 25%; ++, 25-50%; +++, 50-75%; ++++ > 75%.

The immunolocalization in cancer cells was related to the physiological function and potential of Sp17 as a target for *in vivo *imaging diagnosis and immunotherapy of tumors. Its heterogeneous expression was demonstrated in the cytoplasma, nucleus and cell surface. Although Sp17 is not easily identified in the cell membrane of gynecological cancer tissues, its presence can be confirmed in a cultured cell line (Fig 3).

Our previous study revealed that overexpression of Sp17 in the ovarian cancer cell line HO8910 increased cellular migration, suggesting that Sp17 might enhance tumor progression [20]. However, the relationship between Sp17 and the grade of tumors analyzed in this study failed to support this presumption.

The Sp17 targeted immunotherapy of tumors remains controversial and needs more accurate validation. Those in favor hold that although tumor specificity is an advantage of the therapy, it is extremely difficult to find a molecule absolutely specific for tumor vaccines. Moreover, both cancer patients and oncologists are familiar with and accept the nonspecific toxicities of other anti-tumor therapies [32,33]. In this regard, this protein may be a candidate for a gynecological cancer vaccine. However, in view of the extensive presence of this molecule in somatic cells, further research to evaluate the suitability of Sp17 as a human therapeutic antigen is imperative before it is used as a molecular target for the treatment of tumors.

## Competing interests

The authors declare that they have no competing interests.

## Authors' contributions

FQL conceived, coordinated and designed the study and contributed to the acquisition, analysis and interpretation of data and drafted the manuscript. QL and YLH performed the experiment and were involved in drafting the article. BW and HLY selected the archived samples and scored the immunohistochemistry staining. All the authors have read and approved the final manuscript.

## Pre-publication history

The pre-publication history for this paper can be accessed here:

http://www.biomedcentral.com/1471-2407/10/429/prepub
